# Complete Genome Sequence of *Burkholderia* sp. Strain THE68, a Bacterial Symbiont Isolated from Midgut Crypts of the Seed Bug Togo hemipterus

**DOI:** 10.1128/MRA.00041-20

**Published:** 2020-03-05

**Authors:** Kazutaka Takeshita, Seonghan Jang, Yoshitomo Kikuchi

**Affiliations:** a Faculty of Bioresource Sciences, Akita Prefectural University, Akita City, Akita, Japan; b Graduate School of Agriculture, Hokkaido University, Sapporo, Hokkaido, Japan; c Bioproduction Research Institute, National Institute of Advanced Industrial Science and Technology (AIST), Hokkaido Center, Sapporo, Hokkaido, Japan; d Computational Bio Big Data Open Innovation Laboratory (CBBD-OIL), AIST, Hokkaido Center, Sapporo, Hokkaido, Japan; Indiana University, Bloomington

## Abstract

*Burkholderia* sp. strain THE68 is a bacterial symbiont isolated from the midgut crypts of a phytophagous stink bug, Togo hemipterus. Here, we report the complete 7.98-Mb genome of this symbiont, which consists of six circular replicons containing 7,238 protein coding genes.

## ANNOUNCEMENT

Many species of phytophagous stink bugs belonging to the superfamilies Coreoidea and Lygaeoidea, and members of the family Largidae in the superfamily Pyrrhocoroidea, develop specialized crypts at the posterior region of the midgut, the lumen of which is densely colonized by beneficial bacterial symbionts of the genus *Burkholderia sensu lato* (1, 2). We have reported the two complete genomes of *Burkholderia* symbionts isolated from the bean bug Riptortus pedestris (Coreoidea: Alydidae) (3, 4). Here, we report a newly sequenced complete genome of a *Burkholderia* symbiont, strain THE68, whose host stink bug is phylogenetically different from the bean bug.

The *Burkholderia* symbiont strain THE68 was isolated from the midgut crypts of a wild-captured Togo hemipterus (Lygaeoidea: Rhyparochromidae) in our previous study (5). Genomic DNA was extracted from an overnight culture in yeast-glucose medium (0.5% yeast extract, 0.4% glucose, and 0.1% NaCl) at 28°C using the phenol-chloroform method. A DNA library for Illumina short reads (mean insert size, 500 bp) was constructed by using a Covaris S2 instrument and a HyperPrep kit (Kapa Biosystems). A g-TUBE (Covaris) and a ligation sequencing kit (Oxford Nanopore Technologies) were used for the library construction for Nanopore long reads (mean insert size, 10 kbp). The genome sequencing was performed with NextSeq (Illumina) using the 2 × 151-bp protocol and GridION using an R9.4.1 flow cell (Oxford Nanopore Technologies). The Illumina short reads were processed with Sickle v1.33 (6) to remove the low-quality bases (quality value [QV], <20) and shorter reads (<127 bp). After processing the Nanopore long reads with Porechop v0.2.3 (https://github.com/rrwick/Porechop) and Filtlong v0.2.0 (https://github.com/rrwick/Filtlong) with the options “–min_length 1000 –target_bases 800000000,” error correction was performed by using fmlrc v1.0.0 (7) with the processed Illumina short reads. These processed short and long reads (1.1 Gb and 0.8 Gb, respectively) were assembled by using Unicycler v0.4.7 (8), resulting in six circular replicons. The quality of the assembly was assessed with CheckM v1.0.12 (9) and Bandage v.0.8.1 (10). Each replicon was assigned as a chromosome or plasmid based on comparison with the genomes of B. insecticola RPE64 (3, 11) and B. cordobensis RPE67 (4). The assembled genome was annotated by using DFAST v1.2.4 (12). The value of pairwise digital DNA-DNA hybridization was calculated with GGDC v2.1 (13). All bioinformatic analyses were performed with default parameters unless otherwise indicated.

The complete genome of strain THE68 is 7,982,451 bp (coverage, 234×; GC content, 63.1%; completeness, 99.60%; contamination, 2.03%) and consists of six circular replicons containing 7,238 protein coding genes, 16 rRNAs, and 68 tRNAs. Genome comparisons with other *Burkholderia* symbionts (3, 4) suggested that four replicons are chromosomes and two are plasmids, although the assignments should be carefully confirmed in further studies. The values of pairwise digital DNA-DNA hybridization against other *Burkholderia* symbionts were 31.3% for *B. insecticola* RPE64 (3) and 33.3% for *B. cordobensis* RPE67 (4). According to the proposed criterion based on genomic data (14), strain THE68 isolated from *T. hemipterus* is a different species than the two *Burkholderia* symbionts isolated from *R. pedetris*. More detailed comparisons between these *Burkholderia* symbionts, as well as nonsymbiotic environmental species, enable us to genetically characterize the beneficial *Burkholderia* symbionts of phytophagous stink bugs.

### Data availability.

The genome sequence of *Burkholderia* sp. THE68 has been deposited in DDBJ/ENA/GenBank under accession no. AP022315 through AP022320. The versions described in this paper are the first versions, AP022315.1 through AP022320.1. The raw sequences have been deposited in the DDBJ Sequence Read Archive under accession no. DRA009416.
